# Chancen und Grenzen digitaler Lehre an Hochschulen aus Studierendenperspektive: Empirische Befunde und Gestaltungshinweise

**DOI:** 10.1365/s40702-021-00796-y

**Published:** 2021-09-27

**Authors:** Gergana Vladova, André Ullrich, Benedict Bender

**Affiliations:** 1 Lehrstuhl für Wirtschaftsinformatik, Prozesse und Systeme, Universität Potsdam, Potsdam, Deutschland; 2 Weizenbaum-Institut für die vernetzte Gesellschaft, Berlin, Deutschland

**Keywords:** Digitale Lehre, Covid-19, Digitalisierung, Lehr-Lern-Transaktionsmodell, Gestaltungshinweise, Digital teaching, Covid-19, Digitalization, Teaching-learning transaction model

## Abstract

Die Ausgestaltung qualitativ hochwertiger Lehre erfordert eine Zusammenwirkung zwischen Lehrenden und Lernenden. Die Präsenzlehre profitiert hierzu von einer langjährigen Tradition, digitale Lehre dagegen befindet sich vergleichsweise noch am Anfang ihrer Verbreitung. Ein großer Entwicklungsschritt Richtung Digitalisierung des Unterrichts wurde im Kontext der Hochschullehre während der Covid-19-Pandemie im Frühjahr 2020 erzielt, als der Präsenzunterricht für Monate unterbrochen wurde. Dabei konnten wichtige Erkenntnisse zu Chancen und Grenzen digitaler Lehre gewonnen werden. Dieser Beitrag zeigt ausgewählte Ergebnisse einer Studie, die an vier deutschen Hochschulen und mit 875 Antworten im Frühjahr 2020 durchgeführt wurde. Die Studie deckt Chancen und Grenzen digitaler Lehre aus der Sicht der Studierenden und vor Hintergrund ihrer Erfahrung im komplett digitalen Semester auf. Die Ergebnisse werden als Grundlage für die Ableitung von Gestaltungshinweisen für digitale Lehr- und Lernangebote genutzt. Ausgehend von einem Modell zur Analyse der Ausgestaltung von Lehr- und Lern-Formaten, werden diese Hinweise nach den Elementen Lernende, Lehrende, Lehrinhalte, Umgebung und Lehrstil strukturiert.

## Abstract (en)

The design of qualitative, excellent teaching requires collaboration between teachers and learners. For this purpose, face-to-face teaching benefits from a long-standing tradition, while digital teaching is comparatively still at the beginning of its dissemination. A major developmental step toward the digitization of teaching was achieved in the context of university teaching during the Covid 19 pandemic in spring 2020, when face-to-face teaching was interrupted for months. During this time, important insights into the opportunities and limitations of digital teaching were gained. This paper presents selected results of a study conducted at four German universities and with 875 responses in spring 2020. The study uncovers opportunities and limitations of digital teaching from the students’ perspective and against the background of their experience in the completely digital semester. The results are used as a basis for deriving design guidelines for digital teaching and learning offerings. Based on a model for analyzing the design of teaching and learning formats, these indications are structured according to the elements learners, teachers, teaching content, environment and teaching style.

## Einleitung

Über die vergangenen 15 Jahre stieg der Grad an Digitalisierung in der Lehre rasant an. Anwendung fanden E‑Learning-Plattformen zur Organisation der Lehre sowie virtuelle Kursformate zur Vermittlung von Wissen. Weiterhin wurden zunehmend auch Prüfungsformate digital durchgeführt. Obwohl digitale Bildungstechnologien schon seit Jahrzehnten in der Entwicklung sind, haben diese noch keine nachhaltig transformierende Wirkung auf den Bildungssektor ausgeübt. Der Ausbruch der Covid-19-Pandemie wirkte jedoch als Katalysator für die vorhergehenden Entwicklungen. Der Universitätsbetrieb wurde digital aufrechterhalten und wichtige Erfahrungen über Lehren und Lernen mit digitalen Medien konnten gesammelt werden.

Zu Beginn der Covid-19-Pandemie wurden sofortige Maßnahmen getroffen, um die Verbreitung des Virus zu bekämpfen. Als Folge dessen wurde unter anderem der Präsenzunterricht weltweit unterbrochen (Crawford et al. 2020). Somit wurde ein Prozess über Monate unterbrochen, der nicht nur durch Weitergabe von Wissen sondern auch durch soziale, zwischenmenschliche Beziehungen geprägt ist. Die Hochschulbildung wurde von der Pandemie radikal betroffen (Nuere und de Miguel 2020; Watermeyer et al. 2020). Während des Lockdowns mussten Bildungseinrichtungen ihre Aktivitäten sofort vom Klassenzimmer und dem Campus in eine virtuelle Umgebung verlagern, was die einzige Alternative zu einer vollständigen Handlungsunfähigkeit war (Crawford et al. 2020; Kamarianos et al. 2020; Karalis und Raikou 2020; Owusu-Fordjour et al. 2020).

Diese enorme Herausforderung für die Bildungsinstitute hat ebenso eine Möglichkeit eröffnet, einen großen Schritt Richtung Digitalisierung des Unterrichts zu erzielen und zu wichtigen Erkenntnissen zu gelangen. Es wurde beispielsweise festgestellt, dass nach Monaten online-Unterricht die Studierenden persönliche Treffen mit den Dozierenden vermehrt einforderten (Vladova et al. 2021). Einerseits konnte die Digitalisierung als Katalysator für die Nutzung von Lehr- und Lernplattformen genutzt werden. Die Organisation der Lehreinheiten und entsprechender Materialien sowie Abgaben und auch Prüfungen brachte kurzzeitig sowohl Lehrende als auch Lernende teilweise an ihre Grenzen, langfristig gesehen erscheint diese Erfahrung jedoch als großer Mehrwert für die Gestaltung digitaler Lehre. Es konnte festgestellt werden, dass einzelne Formate, die im klassischen Frontalunterricht durchgeführt werden, auch in nicht-physischer Präsenz in geforderter Qualität durchführbar sind, andererseits lassen sich bestimmte Lehr- und Lerninhalte nicht digital vermitteln. Das deutet unter anderem eine zukünftige Tendenz zu hybriden Lehr- und Lernformaten an. Insbesondere bei künstlerischen Fachrichtungen zeigte sich eine leibliche Co-Präsenz als unabdingbar.

Die langfristigen Auswirkungen dieses Einschnitts bei Lehr- und Lernformaten hin zur kompletten Digitalisierung der Lehre bleiben jedoch vorerst noch unklar. Um einen Beitrag auf dem Weg zur Beantwortung der Auswirkungen zu leisten, werden in diesem Beitrag die Einschätzung der Studierenden, als Kernzielgruppe der digitalen Lehre in Universitäten, hinsichtlich der vollständig digitalen Lehrperiode untersucht. Dabei stehen insbesondere deren Akzeptanz hinsichtlich dieses Formats sowie deren Wahrnehmung der Lehr- und Lernplattformen im Fokus.

Mittels einer Umfrage an deutschen Hochschulen in der Zeit des ersten Lockdowns (Frühling/Sommer 2020) wurde in dieser Studie mit offenen und geschlossenen Fragen die Einstellung der Studierenden zur digitalen Lehre ermittelt. Ausgehend von den Ergebnissen dieser Umfrage werden im Beitrag Gestaltungshinweise und Erfolgsfaktoren für digitale Lehrformate beschrieben. Wir beleuchten aus Perspektive der Studierenden ebenso die größten Hürden bei der Digitalisierung der Lehre sowie das optimale Verhältnis zwischen Offline- und Online-Lehre. Weiterhin gehen wir auf die wahrgenommenen Grenzen der Wissensvermittlung, sozialer Interaktion und Lehrgestaltung bei digitalen Lernangeboten ein. Das Ergebnis bilden Handlungsempfehlungen zur Verbesserung der Qualität digitaler Lehre an Hochschulen.

## Das Lehr-Lern-Transaktionsmodell – eine Strukturierungsgrundlage für den (digitalen) Unterricht

Qualitativ hochwertige Lehre ist eine gemeinsame Erfahrung von Lehrenden und Lernenden im Lernprozess, wobei beide voneinander lernen (Dees et al. 2007). Zu betonen ist der transaktionale Charakter der Erfahrung von Qualitätsunterricht, wobei Lernende und die Interaktion mit ihnen als sehr wichtig für die Qualität und Verbesserung des Lehrprozesses gesehen werden. Lernende bringen „ihre eigenen Auffassungen und Erwartungen an das Lehren und Lernen mit, die mit den Auffassungen und Erwartungen des Lehrers in Konflikt stehen, sie ergänzen oder sich überschneiden können.“ (ebd. S. 131).

Das Lehr-Lern-Transaktionsmodell (vgl. auch im Folgenden Dees et al. (2007)) kann als Leitfaden zur Reflexion vor, während und nach einer Lehrveranstaltung verwendet werden und bietet ein Instrumentarium zur Identifikation und Analyse der Ausgestaltung von Lehr- und Lern-Formaten sowie -Settings. Es kann weiterhin als Leitfaden für die Planung von Lernaktivitäten genutzt werden.

**Abb. 1:**
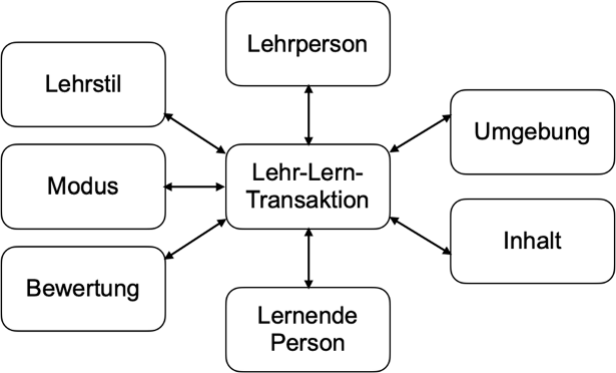
Lehr-Lern-Transaktionsmodell. (Dees et al. 2007)

Die Hauptelemente des Modells sind in Abb. 1 dargestellt. Die **Lehrperson** ist der selbstreflexive Teil des Modells. Dieser befasst sich mit den individuellen Charakteristika, den Erwartungen und Überzeugungen der Lehrperson. Die** Lernende Person** ist ein wichtiger Teil der Unterrichtstransaktionen und reflektiert den Lernstill, die Erwartungen der Lernenden, deren Motivation und Fähigkeiten. Der **Lehrstil** adressiert die Erscheinungsform der Lehrperson im Klassenzimmer hinsichtlich Art und Weise, wie auf die Fragen und Anforderungen der Lernenden reagiert und wie der Lehr-Lernprozess organisiert wird. Im Fokus steht das allgemeine zwischenmenschliche Unterrichtsklima, das durch die Handlungen und die allgemeine Persönlichkeit der Lehrkraft entsteht. Der **Modus** zeigt, wie die Lehrperson den Lernprozess gestaltet und welche Art von Aktivitäten geschaffen werden, um den Lernenden Wissen zu vermitteln. Beispiele hierzu sind der Vortrags- vs. Diskussionsmodus oder der problemorientierte Lernmodus. Bei den **Inhalten** geht es sowohl um die eigentlichen Lerninhalte als auch um die relevanten pädagogischen Fragestellungen, die mit dem Lehren verbunden sind. Relevant ist das Zusammenspiel zwischen Inhalt und Lernfeld sowie die Auswirkung dieser auf die individuelle Entwicklung und das generelle Verständnis der Studierenden. Die **Umgebung** adressiert den Raum, in dem der Lernprozess stattfindet – die physische, soziale oder auch virtuelle Umgebung für Lernen und Lehren. Die **Bewertung** umfasst die Art und Weise, wie das Wissen der Lernenden ermittelt wird und wie es sich auf die Unterrichtserfahrung auswirkt. Die Bewertung ist wichtig für die Gestaltung des Lernprozesses: Die Erwartungen der Studierenden, wie sie bewertet werden und welche Kenntnisse und Fähigkeiten sie erwerben, sind entscheidend dafür, wie sie lernen. Weiterhin können Lernende eine gute Bewertung nutzen, um den eigenen Lernprozess besser zu reflektieren.

Diese Elemente können als Ausgangspunkt für die Betrachtung und Analyse von Lehr- und Lehrsituationen verwendet werden und – im Sinne des vorgestellten Transaktionsmodells – Lehrkräfte bei der Gestaltung des Unterrichts zu unterstützen. In diesem Beitrag werden die Elemente zur Strukturierung der Analyse der Antworten der Studierenden in der Studie genutzt, mit dem Ziel, die Qualität des digitalen Lehr- und Lernsettings im komplett digitalen Modus während des Lockdowns zu bewerten und so strukturiert Handlungsempfehlungen für die Digitalisierung der Hochschulbildung zu generieren.

## Studiengestaltung und -verlauf

Die diesem Beitrag zugrunde liegende Datenbasis wurde in einer Längsschnittstudie erhoben, die als Online-Umfrage durchgeführt wurde. Während der Vorlesungszeitraums des ersten COVID-19-Semesters (April bis Juli 2020) wurden Studierende zu vier verschiedenen Zeitpunkten (monatlich) hinsichtlich ihrer Wahrnehmung des digitalen Lernens befragt. Die Umfrage wurde an vier deutschen Universitäten jeweils in den gleichen Lehrveranstaltungen durchgeführt. In Universität 1 haben Masterstudierende der Wirtschaftsinformatik (WI) teilgenommen, in den übrigen drei Universitäten wurden Studierende der Fachrichtungen Musik und Kunst befragt. Es wurden keine Daten zu einer bestimmten Lehrveranstaltung erhoben, sondern zur Lehrsituation im digitalen Semester im Allgemeinen. Eine Besonderheit bieten digitale Einzelunterrichtsangebote bei den künstlerischen Fächern, hierzu wurde jedoch lediglich nach einer Einschätzung der Studierenden zu der Qualität dieser Unterrichtseinheiten gefragt, somit sind die Antworten für diesen Beitrag nicht relevant.

Zentrales Erkenntnisinteresse der Umfrage war es, die Akzeptanz gegenüber digitaler Lehre durch Studierende zu erheben und weiterhin konkrete Vor- und Nachteile des digitalen Lernens zu erfassen. Dies wurde mittels vordefinierter Frage- und Antwortkategorien erhoben. Die Studie nutzte folgende theoretische Konstrukte als Grundlage: (1) Studien zur (Technologie‑)Akzeptanz von E‑Learning und (2) Studien zu den Vor- und Nachteilen von E‑Learning im Vergleich zu Face-to-Face- oder Blended Learning. Es wurden, mit kleinen Modifizierungen, vorab getestete Skalen bei der Umfrage genutzt. Details zur Erhebungsmethodik, den genutzten Skalen sowie dem Fragebogen sind in Vladova et al. (2021a) zu finden.

Darüber hinaus wurden demografische Daten erhoben und offene Fragen gestellt, um weitergehende Einblicke in die Wahrnehmungen der Studierenden im Laufe des Semesters zu gewinnen. Die offenen Fragen wurden gemeinsam mit Dozierenden an den untersuchten Hochschulen entwickelt und hatten als Ziel, weitere Einblicke in die Einschätzung der Studierenden zu gewinnen.

Im Rahmen der Umfrage wurden 875 Antworten gesammelt, wovon 246 (28 %) von Studierenden der Fachrichtung Wirtschaftsinformatik und 629 (72 %) der Fachrichtungen Kunst und Musik enthalten waren. Hinsichtlich der zeitlichen Verteilung, wurden 147 Antworten im April, 319 im Mai, 269 im Juni und 128 im Juli gesammelt. Davon entfallen 59 % (513) auf Frauen und 35 % (310) auf Männer. Der Rest machte keine Angabe oder wählte „sonstiges Geschlecht“ als Antwortmöglichkeit aus.

Die Grundlage für die nachfolgende Auswertung bilden die kombinierten Rückmeldungen aus allen Erhebungszeitpunkten (*n* = 875). Für eine Auswertung der Daten nach den unterschiedlichen Phasen sowie in Bezug auf die Besonderheiten der unterschiedlichen Studienfächer sei auf Vladova et al. (2021a) verwiesen. Durch die Kombination der Antworten können über unterschiedliche Phasen hinweg Eindrücke und Bedarfe der Studierenden berücksichtigt werden. Im Mittelpunkt dieses Beitrags steht die Auswertung insbesondere der folgenden (offenen) Fragen: Welche Aspekte der Präsenzlehre vermissen die Studierenden am stärksten? Welche Aspekte der Lehre lassen sich aus Sicht der Studierenden nicht zufriedenstellend digitalisieren? Die Antworten dieser Fragen wurden mithilfe eine Inhaltsanalyse untersucht und wiederholende zentrale Aussagen zusammengefasst. Zusammen mit den Ergebnissen anderer geschlossener Fragen der Studie werden diese nachfolgend vorgestellt und diskutiert, um zentrale Hindernisse auf dem Weg zur Digitalisierung zu identifizieren, sowie die Wahrnehmungen der Studierenden von Lehrpersonen, Inhalten, Lernenden und zur technischen Umgebung vorzustellen und zu diskutieren.

## Chancen und Grenzen der Hochschulbildung durch Digitalisierung

### Stolpersteine und optimales Verhältnis zwischen digitalem und Präsenzunterricht

In der Studie wurde die Meinung der Studierenden zu möglichen Stolpersteinen für die Digitalisierung der Lehre erhoben, um Hindernisse für die nachhaltige Verankerung digitaler Lehrangebote in der Hochschulbildung zu identifizieren. Da es sich bei der universitären Lehre um einen formal organisierten Bildungsprozess handelt, war es wichtig, eine Einschätzung darüber zu bekommen, inwieweit **organisationale Aspekte** die Digitalisierung der Lehre ermöglichen oder diese hindern, bzw. als Stolperstein angesehen werden. Weiterhin wurde die **Akzeptanz der Schlüsselakteure – Lehrende und Lernende** – bezüglich der Digitalisierung der Lernprozesse abgefragt. Die **technischen Voraussetzungen** bildeten die vierte Kategorie, die zur Auswahl stand. Da digitale Lehre eine andere Form der Interaktion voraussetzt als Präsenzunterricht, wurde in der Studie weiterhin danach gefragt, inwieweit **die unterschiedlichen Voraussetzungen zur sozialen Interaktion** als ein Stolperstein von den Studierenden wahrgenommen werden.

**Abb. 2:**
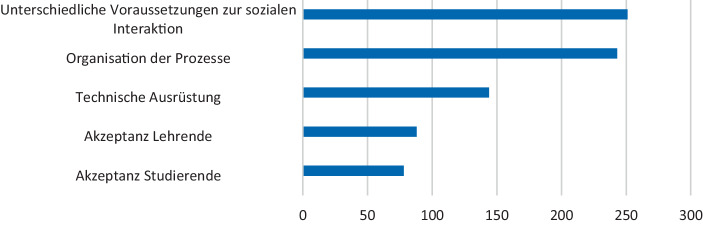
Stolpersteine für die Digitalisierung der Lehre aus Sicht der Studierenden

Die Antworten der Studierenden zeigen, dass die Organisation der Prozesse sowie die Veränderung der sozialen Interaktion als sehr kritisch eingeschätzt werden (Abb. 2). Unproblematisch dagegen ist die Akzeptanz der Studierenden sowie der Lehrenden bezüglich der Digitalisierung der Bildungsprozesse an der Heimatshochschule. Die technischen Voraussetzungen zur Realisierung dieses Wandels wurden von weniger als einem Viertel der Befragten als kritisch betrachtet.

Weiterhin wurden die Studierenden nach ihrer Meinung gefragt, wie das optimale Verhältnis zwischen digitalem und Präsenzlernen (in Prozent) ist. Die Mehrheit der Befragten (47 %) bestimmten den optimalen Anteil digitaler Lehre im Bereich zwischen 10 % und 50 %, jedoch waren weitere 37 % der Studienteilnehmer*innen überzeugt, dass der digitale Anteil zwischen 50 % und 90 % sein sollte. Lediglich 3 % wünschten komplett digitale Lehre, 8 % waren für einen Anteil von weniger als 10 %.

### Lehrperson – Lehrstill, gewählter Modus, Umgebung und Bewertung

Im Mittelpunkt der Studie stand die Perspektive der Studierenden, aus diesem Grund wurde die Meinung der Lehrenden nicht erhoben. Die Antworten der Studierenden wurden benutzt, um ihre Wahrnehmung bezüglich der Gestaltung des Unterrichts durch die Lehrenden zu erfassen. Es wurden konkrete Fragen zum Modus, Lehrstil und Beurteilung gestellt. Es wurde erhoben, ob Studierende darauf vertrauen, dass die für sie zuständigen Lehrpersonen für ihre Inhalte passende: 1) didaktisches Konzept (**Lehrstil**), 2) technische Lösung (**Modus und Umgebung**) sowie 3) Assessmentkonzept (**Bewertung**) ausgewählt haben. Weiterhin hatten die Befragten die Möglichkeit, offene Fragen hierzu zu beantworten.

**Abb. 3:**
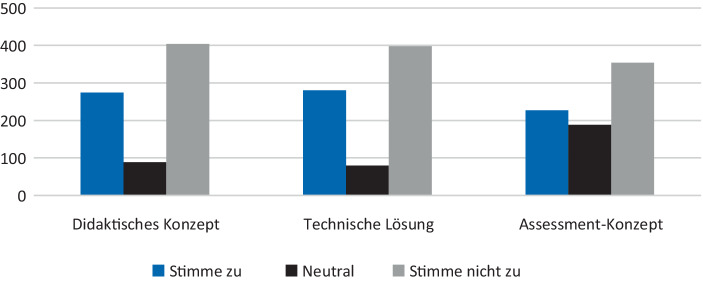
Vertrauen gegenüber der Lehrperson (Wahl des Lehrstils, des Modus, der Bewertung) (Zustimmung zur Aussage: „Ich habe volles Vertrauen, dass meine Dozenten die richtige Entscheidung getroffen haben.“)

Die Ergebnisse zeigen, dass unter den Studierenden keine eindeutige Meinung darüber vorherrscht, ob die von den Lehrenden ausgewählte technische Lösung sowie das didaktische und das Prüfungskonzept passend für die jeweilige Veranstaltung sind. In allen drei Bereichen überwiegt jedoch eine positive Einstellung. Die Studierenden gaben häufiger an, dass sie zuversichtlich sind, die Lehrpersonen haben die passende Lehr- und Assessmentumgebung geschaffen. Bei der Frage nach der Einschätzung, ob das Assessment ihrer Leistungen passend ausgewählt wurde, hat der Großteil der Studienteilnehmer*innen zustimmend geantwortet. Jedoch hatte ein Viertel der Teilnehmer*innen keine Meinung geäußert (vgl. Abb. 3). Eine mögliche Erklärung dafür ist, dass zum Erhebungszeitpunkt noch keine Erfahrung mit digitalen Prüfungen vorhanden war.

Weitere Erkenntnisse, insbesondere zum Lehrstil im digitalen Modus sowie zur gewählten technischen Umgebung konnten aus den offenen Fragen gewonnen werden. Diese werden nachfolgend zusammengefasst vorgestellt mit repräsentativen Beispielaussagen von Umfrageteilnehmern zur Verdeutlichung. So konnten nach Aussagen der Studierenden Lehrende im Rahmen des Unterrichts *„aufgrund der schlechten Audioqualität leider nicht auf die Feinheiten eingehen und diese nicht korrigieren oder Verbesserungsvorschläge geben“*. Negativ aufgefallen sind auch die Ablenkung und die Verständnisprobleme durch Störgeräusche und schlechte Verbindung bei Lehrenden und Lernenden. Weiterhin kritisierten Studierende vermehrt die *„Zeitverschwendung durch technische Probleme und Fragen“*, die als Folge hatte, dass *„häufig nicht alle Fragen wahrgenommen und beantwortet“* werden. In den Lehreinheiten im digitalen Modus überwog der theoretische Anteil sowie die *„formalen Aufgaben zur Vorbereitung auf die Praxis, die dann stattfindet, wenn es wieder geht. Texte lesen und über Zoom in Austausch über die Inhalte kommen“*. Die Dozierenden gestalteten den Unterricht mit *„viel Eigenarbeit, die auch nur theoretisch stattfinden kann, aber auf die praktischen Aufgaben im Herbst vorbereitet“*. Kritisiert wurde der Austausch mit den Lehrenden und die Gestaltung der Möglichkeiten hierzu: „Viele Dozent*innen haben kaum gute Strukturen in den Moodle-Kursen, was oft verwirrend ist. Andere sind nicht erreichbar, was auch für zusätzlichen Stress sorgt. Besonders in den künstlerischen Unterrichten fehlt der aktive Austausch, welcher auch meistens durch Videokonferenzen nicht ersetzt werden kann“. Auch *„die Stunden wurden wesentlich reduziert (maximal alle zwei Wochen)“* und der Unterricht bestand häufig aus „*Hin- und Herschicken von Audiodateien oder Videos, (..) manchmal digitales Treffen über Skype oder Zoom für Rückmeldungen“*. Die Auflösung der formalen Unterrichtsstruktur, mit formaler Zeit- und Ortgebundenheit, wurde als störend empfunden: *„Seminare, die wiederum nur per E‑Mail absolviert werden, sind eine absolute Katastrophe. Man schreibt aneinander vorbei, für Kleinigkeiten will man keine E‑Mail schreiben, Doz[ierende] melden sich sehr unregelmäßig und es wird ohne Deadline gearbeitet. Ob ich auf die E‑Mail sofort antworte und die Aufgabe bearbeite oder erst in einer Woche, scheint irrelevant zu sein. Die Motivation wird dadurch in den Boden gestampft“.*

Es gab auch positive Rückmeldungen, die insbesondere die Wichtigkeit zum Ausdruck brachten, in dieser Ausnahmesituation den Unterricht fortführen zu können und nicht (komplett) zu unterbrechen: *„Es klappt sehr viel besser als erwartet. Mein Dozent kann mir natürlich nicht das gleiche vermitteln, wie im Präsenzunterricht, aber man bleibt am Ball und bekommt viele wichtige Hinweise“.*

Die komplett digitale Unterrichtsumgebung wurde durch den Einsatz digitaler Lernplattformen ermöglicht. Die technologische Unterstützung durch Lernplattformen hat sich positiv auf die Einstellung gegenüber dem digitalen Lernen ausgewirkt, wobei die passende Wahl der Lernplattform zur Zufriedenheit der Studierenden mit digitalem Lernen beigetragen hat. Digitale Lernplattformen bieten ein wesentliches Element zur Strukturierung und Koordination des Lernens sowie der zugehörigen Lerninhalte, wobei laut dieser Studie die adäquate Nutzung der angebotenen Funktionen durch die Lehrenden eine wichtige Voraussetzung für den Erfolg des digitalen Unterrichts bildet.

### Inhalt

Im Mittelpunkt vom Hochschulunterricht stehen sowohl die Übermittlung theoretischer Kenntnisse als auch die Sammlung praktischer Erfahrungen und das selbstorganisierte Lernen.

**Abb. 4:**
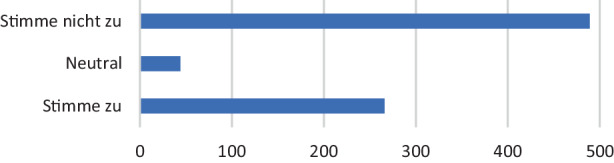
Vermittlung von Lerninhalten im digitalen Unterricht (Zustimmung zur Aussage: „Die Inhalte meines Studiums lassen sich problemlos digital übermitteln“)

Gemäß der Mehrheit der Studierenden sind die Inhalte des Studiums nicht problemfrei digital übermittelbar (vgl. Abb. 4). Dies ist insbesondere wichtig, da die Auswertung der Daten zeigt, dass die Zuversicht über die digitale Vermittelbarkeit der Inhalte des eigenen Studiums Studierende dabei beeinflusst, die Digitalisierung des Hochschulunterrichts im Allgemeinen zu befürworten. Auch die Covid-19-Pandemie (und den vollständig digitalen Modus) sahen Studierende eher als Chance zur Verbreitung digitaler Lehr- und Lernformate an Hochschulen, wenn sie der Meinung waren, die Inhalte ihres Studiums lassen sich problemlos digital vermitteln. Die Datenanalyse deutet darauf hin, dass das Vertrauen der Studierenden zu den vorher vorgestellten drei Elementen im Modell von Dees et al. (2007) – Bewertung, Modus und Lehrstil – positiv damit korreliert, ob die Inhalte des eigenen Studiums aus der Sicht der Befragten unproblematisch im digitalen Unterricht vermittelt werden können.

Bei der Auswertung der offenen Fragen, die teilweise explizit den Aspekt der Digitalisierbarkeit der Inhalte aus Sicht der Studierenden adressierten, wurde deutlich, welche Unterschiede und Defizite erkennbar sind. So sind die Befragten weitgehend zuversichtlich, dass Vorlesungsinhalte – und somit die Vermittlung von Faktenwissen – problemlos in den digitalen Raum übertragbar sind. Die Inhalte, die weniger geeignet für den digitalen Bereich sind, lassen sich in zwei Hauptbereiche unterteilen:

- Mit direktem Bezug zum **Unterrichtsinhalt** sehen die Studierenden allgemein für alle Fachbereiche als problematisch die Digitalisierung von: *„Gruppenunterricht, Proben, Aufführungen, Projektbetreuung Pausenaustausch“, „Präsenzgruppenarbeiten und dynamische Austausch und Diskussionen“, „Das !effektive! Lernen: z.* *B. digital in Gruppen Projekte zu planen und durchzuführen“, „Laborexperimente“*. Darüber hinaus bestehen fachspezifische Herausforderungen, z. B. (Fach Musik) „*Über digitale Medien können zu wenig Dimensionen des Lerngegenstandes übertragen werden und es fehlt die direkte Interaktion und Kommunikation auf Klang- und Körperebene, wodurch erst notwendige Erfahrung entsteht“*.
- In Bezug auf die **Zusammenarbeit** wird insbesondere der Austausch und das Feedback von und an Dozierenden durch die Digitalisierung als beeinträchtigt angesehen, wobei Studierende Lernen als *„das Ergebnis einer lernfördernden Lehrer-Schüler-Beziehung“* beschreiben und darauf hinweisen, dass durch die Unzulänglichkeiten bei der Übermittlung von Inhalten über die Technik, Distanz und Kommunikationsprobleme entstehen. Auch *„das direktere Fragenstellen, das Gefühl irgendwo „aufgehoben“ zu sein, wenn man etwas nicht versteht, oder sich unsicher bei etwas ist“* sind im digitalen Raum nicht möglich. Das wird auch als Grund dafür angesehen, dass *„die realistische Einschätzung der Lehrenden über den Arbeitsaufwand für Aufgaben“* fehlt und von Studierenden mehr verlangt wird.

In einer der Antworten wird die Kritik wie folgt zusammengefasst: *„Wer glaubt, dass das Digitale die Zukunft ist bzw. vorangetrieben werden soll, einfach weil es da ist, ohne gewissenhaft reflektiert zu haben, was die Technik mit uns macht, ist ein Narr und naiv“*.

### Lernende Person

Im Modell von Dees et al. (2007) wird darauf hingewiesen, dass die Eigenschaften der Lernenden bei der Gestaltung des Unterrichts berücksichtigt werden müssen. Im digitalen Raum ist es von Bedeutung, dass die notwendigen technischen Voraussetzungen auch bei Lernenden vorhanden sind. Jedoch verfügten 14 % der Befragten über keine stabile Internetverbindung und 20 % waren nicht sicher, ob ihre Verbindung stabil genug ist (*N* = 833). Dies weist darauf hin, dass ein Drittel der Studierenden technische Probleme im Unterricht hatte. Weiterhin ist für das Studium eine geeignete Lernumgebung notwendig. Hierzu bestehen ebenso Defizite bei einem Drittel der Befragten, die angaben, keinen geeigneten Platz zum Lernen zu haben.

Unterschiede bestehen in Bezug auf das Studienfach der Befragten (ausführlich in Vladova et al. (2021a) diskutiert). Generell äußerten sich Kunst und Musik-Studierende zu allen Fragen kritischer als Wirtschaftsinformatik-Studierende, was zu dem Schluss führt, dass sie (vollständig) technologievermittelte Lehre nicht in gleichem Maße akzeptieren wie Wirtschaftsinformatik-Studierende. Letztere haben laut der Ergebnisse der vorliegenden Studie die technologievermittelte Lehre mehr genossen als die anderen zwei Fachgruppen.

Die Beantwortung der offenen Fragen erlaubt weitere Erkenntnisse dazu, was aus Sicht der Lernenden wünschenswert bei der Gestaltung der Lehre ist. Lernende möchten *„noch stärker in den Lernprozess miteinbezogen werden, indem sie aktiver an Lehrveranstaltungen teilnehmen können (kleine Aufgaben, Quizfragen, unmittelbares Feedback). Weiterhin sollten Lernspiele entwickelt werden, die mehr Spaß machen (Lernspiele)“*. Weiterhin soll der Austausch zwischen den Lernenden – fachlich und sozial – gefördert werden, das Studium wird nicht nur als Wissensvermittlung, sondern auch als *„Möglichkeit für soziale Weiterentwicklung“* gesehen. Darüber hinaus wird die Möglichkeit zur sozialen Interaktion als zu eingeschränkt empfunden. Es wird vermisst, *„dass man auch mal privat vor oder nach dem Unterricht mit dem Professor oder Lehrenden quatschten kann unter vier Augen über Dinge, die einem am Herzen liegen“*.

## Gestaltungshinweise zur Durchführung digitaler Hochschullehre

Die gewonnenen Erkenntnisse aus der Analyse der Antworten der Studienteilnehmer*innen werden nachfolgend in Form von Gestaltungshinweisen zusammengefasst (vgl. Tab. 1). Zur Strukturierung dieser wurden die Modellelemente nach Dees et al. (2007) genutzt, wobei die Ergebnisse der Umfrage den Kategorien im Modell zugeordnet wurden. Zusätzlich wurden studienfachspezifische Unterschiede in einer weiteren Kategorie aufgelistet.

**Tab. 1:** Generierte Gestaltungshinweise für die Umsetzung digitaler Lehr‑/Lernformate. (Strukturiert nach Dees et al. (2007))

Kategorie	Gestaltungshinweis
*Lehrperson* (Lernprozess-gestalter*in)	Grad der Notwendigkeit für den Austausch mit Studierenden einschätzen und eigene Verfügbarkeit kommunizieren
	Ziele, Vorgehensweisen, Anforderungen und Assessment an die Studierenden eindeutig kommunizieren
	Ausreichend Raum und Zeit für Feedback an die Studierenden sicherstellen
	Regelmäßig Feedback zur eigenen Leistung sowie Verbesserungsvorschläge einholen und kritisch reflektieren
	Eigene Kompetenzen beim Umgang mit digitalen Medien kritisch überprüfen und ggf. ausbauen
	Didaktisches Konzept und Assessment mit den digitalen Rahmenbedingungen abstimmen
	Die allgemeine und administrative Organisation der Prozesse an der Hochschule im Hinblick auf digitale Unterrichtsmöglichkeiten kritisch überprüfen und bei Bedarf Änderungen anstoßen
*Lernende Person* (Teilnehmer*in am Lernprozess)	Klare Trennung zwischen Lern- und Freizeit beachten und eine klare Lernstruktur auch bei Selbstorganisation behalten und berücksichtigen
	Eigenen Bedarf an einem direkten persönlichen sozialen Austausch mit anderen Studierenden und mit Dozierenden reflektieren und bei Notwendigkeit kommunizieren
	Eigenen Bedarf an rechtzeitigem Feedback von den Dozierenden an diese kommunizieren
	Geeignete Lernumgebung und Internetverbindung am jeweiligen eigenen Lernort sicherstellen
	Selbstbeobachtung und Reflektion üben, um einer Überlastung als Folge permanenter Erreichbarkeit entgegenzuwirken
*Inhalt* *(Lernbotschaft)*	Zusätzlich zu den eigentlichen Inhalten, auch Methoden zur Selbstorganisation als Unterrichtsinhalt berücksichtigen
	Lernziele an digitales Format anpassen, Abweichungen von den Lernzielen im Präsenzunterricht evaluieren
	Zwischen Wissensformen unterscheiden – den Transfer vom expliziten Wissen und stillschweigenden Wissen unterschiedlich und durch angemessene Mediennutzung gestalten
	(Explizierbare) Vorlesungsinhalte digital übertragen (Online-Vorlesungen, Aufzeichnung der Vorlesungsinhalte)
	Alternative Formen zur Vermittlung von Praxiswissen berücksichtigen (insbesondere für Sozialisation und direkten Austausch sorgen)
	Komplett digitalen Unterricht, Präsenzunterricht und hybride Lernformen in Betracht ziehen und je nach Bedarf anwenden
*Umgebung* *(physisch, virtuell)*	Lernplattformen nutzen, um den Lernprozess zu strukturieren und zu unterstützen
	Lernprozess mithilfe von Lernplattformen abwechslungsreich gestalten
	Funktionalitäten digitaler Lernplattformen nutzen, um Lernen durch die Beanspruchung mehrerer Sinne zu vereinfachen und optimieren
	Breakout-Roms zur interaktiven Gruppenarbeit und Sozialisation, insbesondere bei Praxisseminaren und Übungen, verwenden
	Vielseitige Funktionen zur Organisation des Lernens von Lernplattformen wie z. B. Moodle nutzen
	Besonderheiten und Funktionalitäten verschiedener Lernplattformen bei der Organisation und Vermittlung von Lerninhalten berücksichtigen
	Sich als Lehrperson in der Verwendung der Plattform schulen und weiterbilden lassen
	Zusatzfunktionen der Lernplattformen nutzen, um Interaktion zu fördern (z. B. Diskussionsforen) und Feedback zu holen (z. B. gegenseitige Bewertung)
	Inhalte entsprechend der vielfältigen Möglichkeiten des genutzten technischen Systems strukturieren und aufbereiten
*Lehrstil* *(Erscheinung der Lehrperson im Lehrraum)*	Die Möglichkeiten und die Notwendigkeit eines hohen Interaktivitätsgrads im digitalen Lehrraum berücksichtigen und nutzen
	Unterschiedliche Lehr- und Lernformaten verwenden, um Abwechslung im Lernalltag sicherzustellen
	Klare Fristen an die Studierenden kommunizieren
	Klare und nachvollziehbare Strukturierung des Kurses gewährleisten
	Insbesondere im digitalen Frontalunterricht Pausen (z. B. durch remote-energizer) zur physischen und psychischen Auflockerung einbauen
	Gruppenarbeiten ermöglichen, um den notwendigen Interaktionsgrad zu fördern
*Studienfach* *(Fachlicher Hintergrund der Lernenden)*	Vorerfahrungen der Studierenden mit digitalen Medien je nach Studienfach berücksichtigen
	Angemessenheit des gewählten Mediums für die Vermittlung der spezifischen Inhalte eines Studienfachs überprüfen
	Generelle Offenheit der Studierenden aus einem bestimmten Studienfach gegenüber technologievermitteltem Unterricht überprüfen (Akzeptanzanalysen) und berücksichtigen
	Überprüfen, inwieweit Studierende Vertrauen haben, dass der Inhalt ihrer Vorlesung digital vermittelt werden kann
	Akzeptanz der Studierenden bezüglich digitaler Lehre im Allgemeinen und konkret in Bezug auf die eigenen Studienfachinhalten messen und berücksichtigen

## Fazit

Es lässt sich zusammenfassen, dass an den befragten Hochschulen weiterhin dem vorgesehenen Unterrichtsplan gefolgt wurde – im Unterschied zu der Situation an Schulen, wo häufig der Unterricht unterbrochen und alternativ gestaltet wurde. Die Ausgangssituation dafür war jedoch unterschiedlich schwierig und hat insbesondere Lehrende stark herausfordert, didaktisch und methodisch qualitativ hochwertig zu lehren.

Die ermittelten Erkenntnisse weisen darauf hin, dass die Digitalisierung der Lehre an Hochschulen nicht lediglich der Einführung einer bestimmten Technologie in den Klassenraum gleichgestellt wird, sondern mit diversen spezifischen Herausforderungen verbunden ist. Die Erfahrung mit der komplett digitalen Lehre hat gezeigt, dass die Lernenden sowohl Vor- als auch Nachteile im digitalen Unterricht sehen. Auch wenn ihr Vertrauen in das Bildungssystem und insbesondere in die Lehrenden im Allgemeinen hoch ist, lenken die Ergebnisse die Aufmerksamkeit auf die Besonderheiten der zu vermittelten Inhalte (Wissensarten) und auf die spezifischen Merkmale der Lernenden als größte Herausforderungen für den Erfolg des digitalen Unterrichts. Unabhängig davon, ob die Lehrenden didaktisch und methodisch souverän die digitale Lehrumgebung gestalten, werden aus der Sicht der Studierenden nicht alle Inhalte gleichermaßen gut vermittelt. Auch ihre sozialen Bedürfnisse dürfen nicht vernachlässigt werden – die Hochschule wird nicht lediglich als eine Institution der Wissensvermittlung, sondern auch der Persönlichkeitsentwicklung angesehen.

Die Studie hat weiterhin gezeigt, dass die notwendige technische Infrastruktur nicht überall in ausreichendem Maße vorhanden war, was den Lernprozess beeinträchtigt hat. Beispielsweise verzichteten Dozierende im Bereich Musik auf synchrone digitale Lehre und lösten die Herausforderungen der nicht ausreichender Audioqualität, indem sie sich Audioaufnahmen der Studierenden zusenden ließen und auf dieser Basis Feedback geben konnten. Demensprechend betrachten einige Studierende die Digitalisierung mit digitalen Medien in der Hochschullehre als positiv, andere eher als negativ und weitere, wiederum, sehen keine großen Unterschiede zwischen Online- und Offline-Lehre bzw. sind dem gegenüber indifferent eingestellt.

Die Ergebnisse der Studie deuten klar darauf hin, dass maßgeschneiderte Ansätze, die sich im jeweiligen Anteil von Online- und Offline-Lehr- und Lernformaten unterscheiden, für Studierende verschiedener Fächer in Betracht gezogen werden sollten. Langfristig sollte weiterhin nicht nur die direkte Lehrpraxis, sondern auch die allgemeine hochschulspezifische Organisation der Lehrprozesse in Blick genommen werden: Notwendige administrative und organisatorische Veränderungen und eine Reorganisation der (gut etablierten) Praktiken, die Anpassung und Weiterentwicklung des Curriculums sowie eine stabile und vertrauensvolle technologische Infrastruktur erweisen sich als notwendig im digitalen Hochschulkontext. Ebenso wichtig für den Erfolg digitaler Lehre sind die Organisation der Lernerfolgskontrolle sowie die Entwicklung einer neuen Kultur des technologievermittelten Unterrichts, einschließlich einer Netiquette mit Verhaltensnormen und Standards.
